# Comparison of phase-resolved functional lung (PREFUL) MRI derived perfusion and ventilation parameters at 1.5T and 3T in healthy volunteers

**DOI:** 10.1371/journal.pone.0244638

**Published:** 2020-12-30

**Authors:** Julian Glandorf, Filip Klimeš, Andreas Voskrebenzev, Marcel Gutberlet, Lea Behrendt, Cristian Crisosto, Frank Wacker, Pierluigi Ciet, Jim M. Wild, Jens Vogel-Claussen

**Affiliations:** 1 Institute for Diagnostic and Interventional Radiology, Hannover Medical School, Hannover, Germany; 2 Biomedical Research in Endstage and Obstructive Lung Disease Hannover (BREATH), Member of the German Centre for Lung Research (DZL), Hannover, Germany; 3 Department of Radiology and Nuclear Medicine, Erasmus University Medical Center Rotterdam, Rotterdam, The Netherlands; 4 Department of Infection, Immunity and Cardiovascular Disease, University of Sheffield, Sheffield, United Kingdom; Medical University of Vienna, AUSTRIA

## Abstract

**Purpose:**

The purpose of this study is to evaluate the influence of different field strengths on perfusion and ventilation parameters, SNR and CNR derived by PREFUL MRI using predefined sequence parameters.

**Methods:**

Data sets of free breathing 2d FLASH lung MRI were acquired from 15 healthy subjects at 1.5T and 3T (Magnetom Avanto and Skyra, Siemens Healthcare, Erlangen, Germany) with a maximum period of 3 days in between. The processed functional parameters regional ventilation (RVent), perfusion (Q), quantified perfusion (Q_Quant_), perfusion defect percentage (QDP), ventilation defect percentage (VDP) and ventilation-perfusion match (VQM) were compared for systematic differences. Signal- and contrast-to-noise ratio (SNR and CNR) of both acquisitions were analyzed.

**Results:**

RVent, Q, VDP, SNR and CNR presented no significant differences between 1.5T and 3T. Q_Quant_ (1.5T vs. 3T, *P* = 0.04), and QDP (1.5T vs. 3T, *P*≤0.01) decreased significantly at 3T. Consequently, VQM increased significantly (1.5T vs. 3T, *P*≤0.01). Skewness and kurtosis of the Q-values increased significantly at 3T (*P*≤0.01). The mean Sørensen-Dice coefficients between both series were 0.91 for QDP and 0.94 for VDP. The Bland-Altman analysis of both series showed mean differences of 4.29% for QDP, 1.23% for VDP and -5.15% for VQM. Using the above-mentioned parameters for three-day repeatability at two different scanners and field strengths, the retrospective power calculation showed, that a sample size of 15 can detect differences of 3.7% for QDP, of 2.9% for VDP and differences of 2.6% for VQM.

**Conclusion:**

Significant differences in QDP may be related to field inhomogeneities, which is expressed by increasing skewness and kurtosis at 3T. Q_Quant_ reveals only poor reproducibility between 1.5T and 3T. RVent, Q, VDP, SNR and CNR were not altered significantly at the used sequence parameters. Healthy participants with minimal defects present high spatial agreement of QDP and VDP.

## Introduction

The global impact of pulmonary diseases like chronic obstructive pulmonary disease (COPD) and asthma on approximately 400 million people clarifies the urgent need for sensitive methods for early detection, disease monitoring and lung function testing [1, 2]. Functional lung imaging offers promising possibilities, but most of the existing techniques are based on contrast enhancement, additional expensive technical equipment or ionizing radiation [3–6]. Some patient groups are unable to undergo certain techniques based on contrast enhancement or spirometry due to potential allergic reactions or the inability of active participation [7–9]. The pursuit of modern MRI techniques for functional lung proton imaging on a regional scale has led to the development of functional free breathing methods of 1H MRI such as phase resolved functional lung (PREFUL) MRI and similar techniques based on Fourier decomposition (FD) [10–12]. These techniques have already proven their ability to provide information on different functional parameters such as ventilation and perfusion in various diseases and can be used for follow up examinations [13–18]. The radiation free examination during free breathing without the need of contrast enhancement makes PREFUL a valuable method for the majority of patients with chronic lung diseases.

In order to establish the reliability of the method for pulmonary disease monitoring, the comparability of the determined functional parameters has to be evaluated across vendor platforms and field strengths. A previous work already demonstrated the robustness of regional ventilation and unquantified perfusion parameters against T1 shortening by gadolinium-based contrast agents (GBCAs) [19]. Another potential influence on functional parameters could be due to the use of different field strengths and their corresponding sequence parameters. Increasing numbers of 3T systems enter the market, because of the potential increase of signal-to-noise ratio (SNR) and contrast-to-noise ratio (CNR) for most organs when compared to 1.5T [20, 21]. In particular lung imaging could benefit from higher SNR as only low intrinsic signal is available [22]. On the other side, the known susceptibility artifacts caused by multiple air-tissue interfaces may be pronounced and further reduce T2* relaxation time [21, 23] thus potentially degrading SNR. Therefore, the effects of higher field strengths on functional lung parameters need to be examined to guarantee reproducibility between different scanners and scan sites. Bauman et al. recently analyzed the impact of higher magnetic field strength on SNR, CNR and relative functional parameters derived by matrix pencil decomposition using steady state free precession and a transient state spoiled gradient echo sequence [24]. The purpose of this study is to evaluate the influence of different field strengths on SNR, CNR and the functional parameters derived by PREFUL MRI in a cohort of healthy participants using a commercially available spoiled gradient echo fast low angle shot (FLASH) sequence.

## Methods

### Participants

The study was approved by the local ethics committee "Ethikkommission" of the "Medizinische Hochschule Hannover" (Study No. 7740_BO_S_2018). Written informed consent was obtained from all participants. Data sets of 16 healthy subjects were acquired and processed. An unequal field of view was used for 1 female participant, who was then excluded due to incomparability of the resulting SNR. The volunteers needed to be 18 to 60 years of age. Exclusion criteria were general inabilities to undergo MRI (e.g. due to claustrophobia, paramagnetic implants, pregnancy). Participants that had a recent (< 1 month) history of lung disease, a chronic lung disease or a known congenital lung disease were excluded.

### Imaging technique

All subjects were examined at 1.5T and 3T scanners (Magnetom Avanto and Skyra, Siemens Healthcare, Erlangen, Germany) with a maximum period of 3 days in between exams. The PREFUL MRI examinations were performed using a spoiled gradient echo FLASH sequence with the parameters shown in Table 1. TR and TE were minimized in regards to scan time reduction and the MR signal was maximized using the Ernst angle.

**Table 1:** Sequence parameters at 1.5T and 3T.

Sequence Parameter	1.5 Tesla	3 Tesla
Field of view	50 x 50 cm^2^	50 x 50 cm^2^
Matrix-size	128 x 128	128 x 128
Slice thickness	15 mm	15 mm
Echo time	0.82 ms	0.74 ms
Repetition time	3 ms	1.83 ms
Flip angle	5°	4°
Pixel bandwidth	1500 Hz/pixel	1500 Hz/pixel

Parallel imaging with GRAPPA with acceleration factor 2 was performed for both acquisitions. All participants were scanned in head first supine position. At 1.5T, 250 images of a single coronal slice at the level of the carina were acquired in ~48 s during free breathing. At 3T, 500 images of the same location were acquired in ~58 s. All images were interpolated to the final in-plane resolution of 256 x 256 pixels prior to reconstruction. The non-uniform intensity profiles of the applied receiver coils were corrected using the sensitivity profiles of the surface and body coil before PREFUL analysis.

### Postprocessing

**Fig 1:**
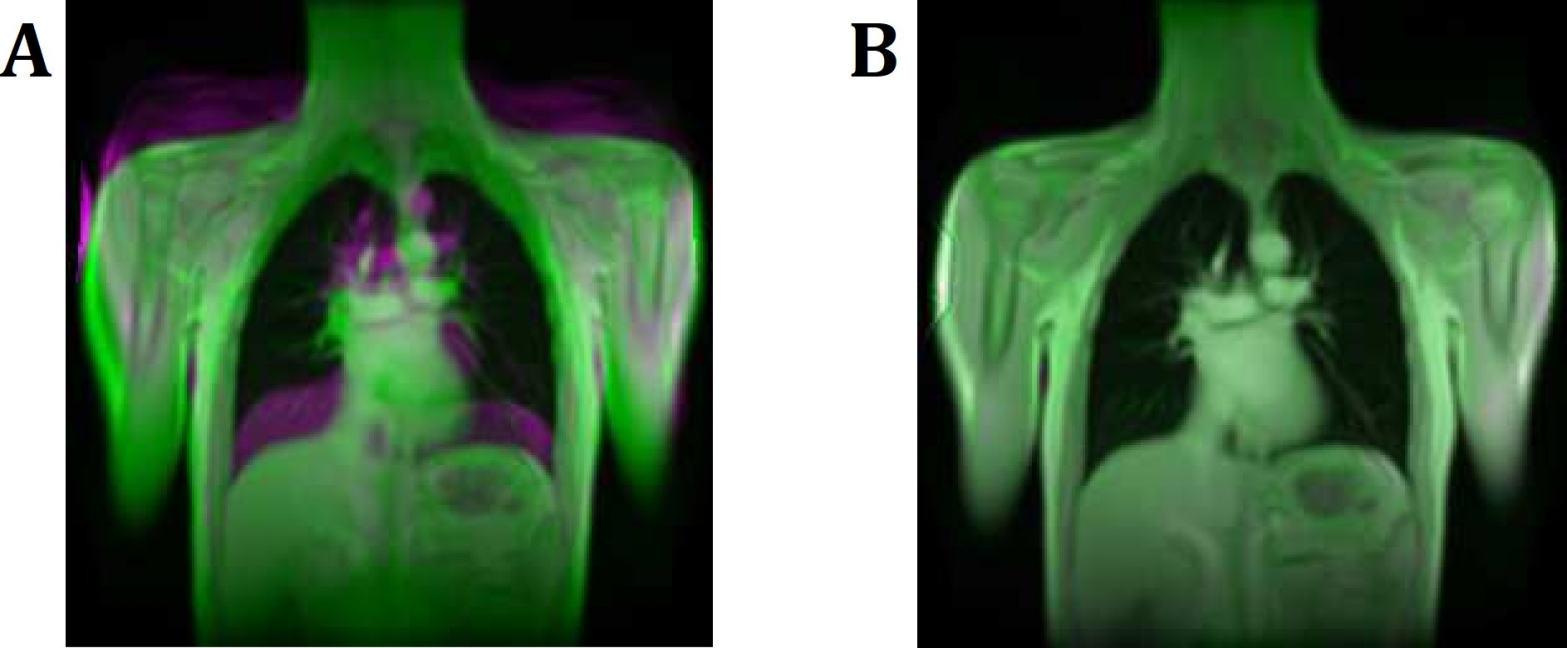
Registration of the 1.5T to the 3T series. Registration between the corresponding 1.5T and 3T series was performed to achieve voxel wise comparability. **A:** Overlay of 1.5T and 3T images before registration. **B:** Overlay image of 1.5T and 3T images after successful registration. Note the increasing structural similarity index (SSIM) of 0.15 before to 0.56 after the registration, which is indicating a successful registration step.

To achieve full saturation and to ensure a steady-state of the stationary tissue, the first 20 images of every PREFUL data set were discarded. All data sets were registered using advanced normalization tools (ANTs) for linear and diffeomorphic image registration with a previously described group oriented registration scheme (GOREG) to achieve a uniform respiratory position for each PREFUL acquisition [25, 26]. In addition, registration between the corresponding 1.5T and 3T series was performed for voxel-wise comparison (see Fig 1). To measure the success of co-registration between 1.5T and 3T, the structural similarity index (SSIM) was calculated [27]. Image guided filtering was applied to all images of every data set using an edge-preserving filter [28].

Each data set was segmented by individual thresholds with the aim to include the vast majority of the lung parenchyma and to exclude large central vessels up to the segmental level. If needed, the segmentations were optimized by manual corrections of insufficiently segmented structures.

**Fig 2:**
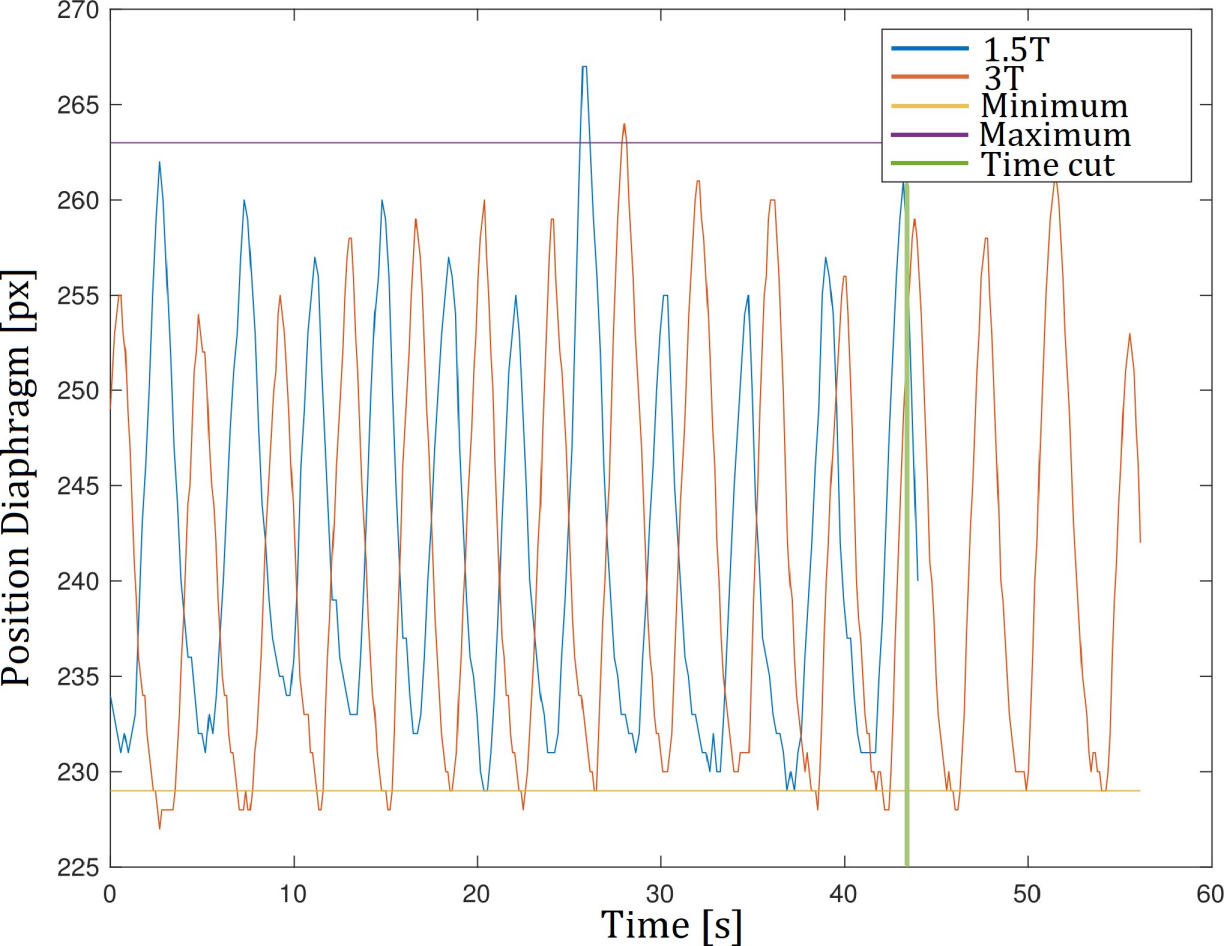
Diaphragm tracking. Respiratory outliers outside the maximum or minimum respiratory level were excluded by removing images outside the intersection of the diaphragm tracking curves of the 1.5T and 3T series. Furthermore, the acquisition time of both series was equalized by truncating the 3T series.

Image outliers due to motion were excluded by removing images outside the intersection of the 1.5T and 3T diaphragm tracking curves. For this, the 3T diaphragm tracking curve was translated onto the 1.5T diaphragm tracking curve by matching the median values of each time series. Furthermore, the total acquisition time of both series was equalized by truncating the 3T time-series (see Fig 2).

### PREFUL ventilation analysis

A low-pass filter at 0.8 Hz was used to remove perfusion effects from the registered images and to calculate ventilation-weighted images. The images were sorted according to their respiratory phase based on the averaged signal amplitude of a region of interest (ROI) positioned at the lung-diaphragm. Subsequently, the sorted images were interpolated to a uniform time grid. Finally, ventilation including phase information was calculated [12]. Regional ventilation (RVent) was used to quantify ventilation according to Klimeš et al. [29]:

$$\text{RVent}=\left(\frac{{\text{S}}_{\text{Mid}}}{{\text{S}}_{\text{Insp}}}\right)-\left(\frac{{\text{S}}_{\text{Mid}}}{{\text{S}}_{\text{Exp}}}\right)$$ (1)

**Fig 3:**
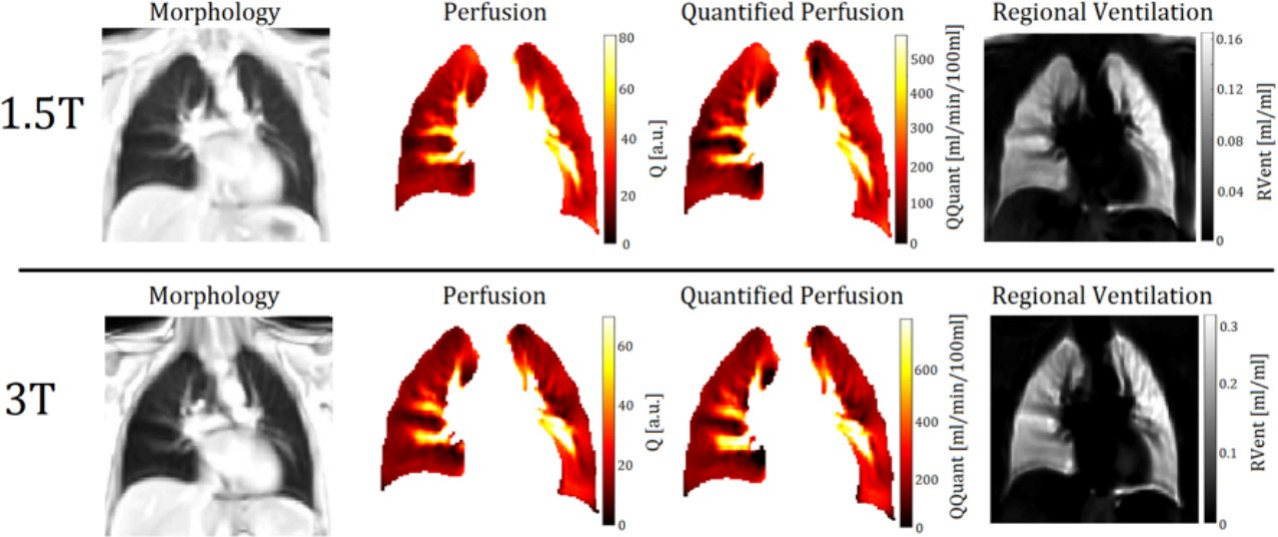
Exemplary PREFUL derived perfusion- and ventilation-weighted maps of a 30-year-old female participant at 1.5T and 3T. Notice the differing scaling.

S_Exp_ and S_Insp_ represent the averaged signals of the end-expiratory and end-inspiratory images. S_Mid_ represents the mean signal of the images in intermediate lung inflation level, which is also used as the reference volume during registration. Ventilation-weighted maps generated at 1.5T and 3T are displayed in Fig 3.

### PREFUL perfusion analysis

A high pass filter at 0.75 Hz was applied to the registered images in order to exclude signal changes due to respiration. Similarly to ventilation analysis, the averaged signal of a manually segmented ROI at the aorta was fitted to a piecewise sine function to estimate the cardiac phase of each time point. Phase information was calculated by sorting the images according to their phase and by interpolating them to an equidistant time grid [12]. Perfusion (Q) was calculated by taking the difference between the signal at the time with the highest and the lowest signal of the perfusion weighted time series in the segmented parenchyma ROI. Perfusion quantification (Q_Quant_) in ml/min/100ml was calculated by normalizing the signal of every voxel to the signal of a completely blood-filled voxel (S_Blood_) according to Kjørstad et al. [30]:

$${\text{Q}}_{\text{Quant}}=\frac{\text{Q}}{{\text{S}}_{\text{Blood}}}*\frac{1}{2*{\text{t}}_{\text{perf}}}$$ (2)

Q represents the perfusion calculated as described above, S_Blood_ represents the corresponding amplitude of the aorta signal (completely blood-filled voxel) and t_perf_ represents the time between two heartbeats. Perfusion- and quantified perfusion-weighted maps generated at 1.5T and 3T are displayed in Fig 3.

### Perfusion and ventilation maps

Perfusion defect percentage maps (QDP maps), ventilation defect percentage maps (VDP maps) and ventilation/perfusion match maps (VQ maps) between each corresponding 1.5T and 3T series were calculated. The upper thresholds for voxels labeled as defect were the median value– 1.75 * standard deviation (SD) for RVent and the median value– 0.75 * standard deviation for Q similarly to Voskrebenzev et al. [12]. The thresholds were set to maintain a VDP < 10% and a QDP < 20% for healthy participants.

### SNR and CNR

Considering the non-central chi distribution in magnitude images of multi-channel receive coils, the SNR was estimated from the ratio of the mean signal in a ROI of the lung parenchyma and the mean signal in a ROI of the background of the image [31]. CNR was calculated by subtraction of the SNR of the parenchyma from the SNR of a lung vessel. Both parameters were calculated for the expiratory and inspiratory phase of each subject at similar ROI positions.

### Statistics

**Fig 4:**
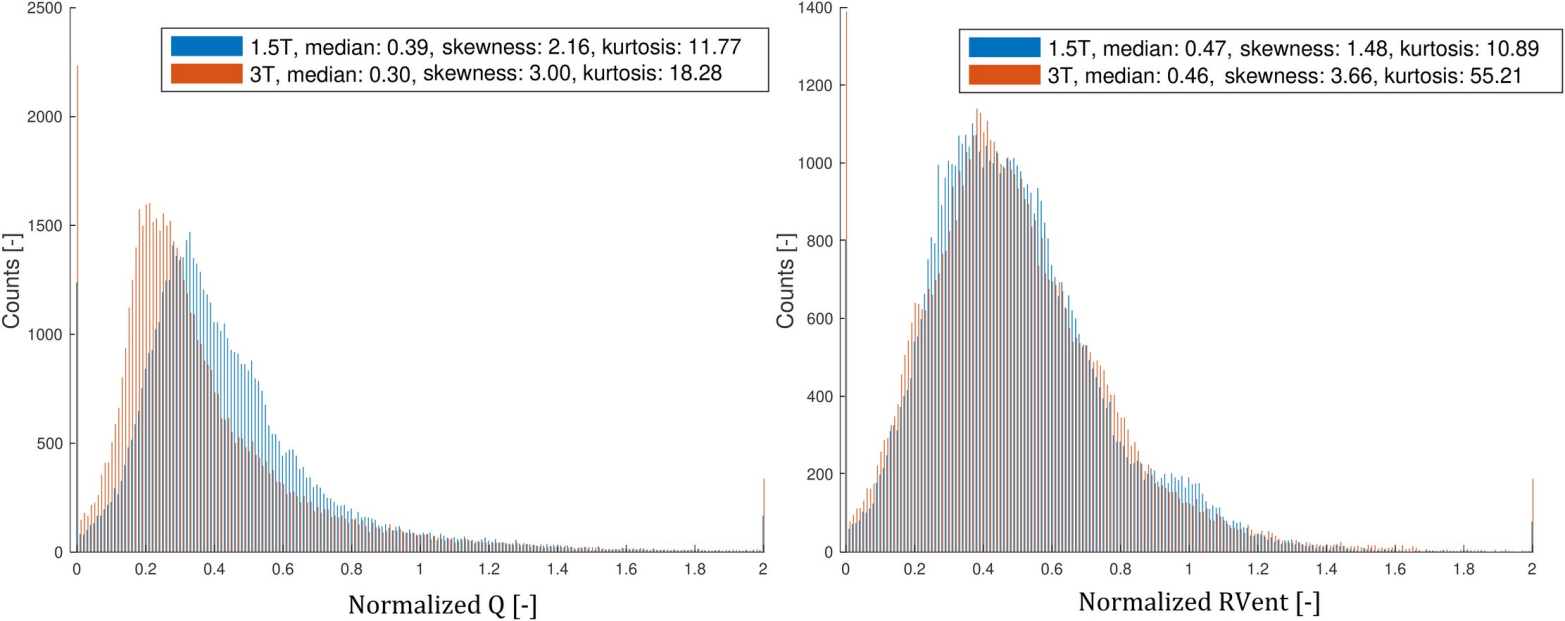
Histogram analysis of the normalized Q- and RVent- values of all participants. The Q- and RVent-values were normalized by division by the 95^th^ percentile of each distribution.

The statistical analysis was performed using JMP Pro 13 (SAS Institute, Cary, NC) and MATLAB R2018a (The MathWorks, Natick, MA). Pretesting with the Shapiro-Wilk test revealed no normal distribution for all parameters. Hence, significant differences of the functional parameters (RVent, Q, Q_Quant_, QDP, VDP and VQ-match percentages) between both corresponding series were assessed by the non-parametric Wilcoxon signed-rank test. Sørensen–Dice coefficients depicting the spatial overlap of QDP und VDP between both series were calculated. Additionally, skewness and kurtosis of the Q- and RVent-values (Fig 4) and the R^2^-fit of the Q-values to the sine function were calculated for both series. Results with *P* < 0.05 were considered as significant. A retrospective sample size calculation and power calculation for three-day repeatability based on the calculated Bland-Altman plots of QDP, VDP, VQM and Q_Quant_ were performed. The calculations are based on a paired t-test using the standard deviations of the mean differences and the mean differences or the number of subjects. The level of significance was set to α ≤ 0.05 and the power level (1-β) to ≥ 0.80.

## Results

### Patient characteristics

The participants included 5 females and 10 males within an age range of 23 years to 55 years and a mean age of 33 years without active smokers. The height ranged from 167 cm to 192 cm with a mean height of 178 cm. The weight ranged from 58 kg to 85 kg with a mean weight of 72 kg. The BMI ranged from 17.96 to 28.06 with a mean value of 22.82.

### Functional parameters, histogram analysis and SNR/CNR

Co-registration was successful for all participants with a mean structural similarity index (SSIM) value of 52.52. The median functional parameters of both series are presented in Table 2. No significant changes were observed for RVent, Q and VDP. On the other hand, Q_Quant_ (339 ml/min/100 ml vs. 246 ml/min/100 ml) and QDP (8.15% vs. 5.04%) decreased significantly at 3T. According to the decrease of QDP and VDP, the VQM increased significantly at 3T (88.78% vs. 91.82%).

**Table 2:** Functional parameters of all participants.

	Functional parameters			Wilcoxon signed-rank test
	1.5 Tesla	3 Tesla	Difference	*P*-value
RVent (ml/ml)	0.14 (0.10;0.16)	0.18 (0.14;0.27)	+ 0.04 (+ 29%)	0.06
Q (a.u.)	21.97 (16.95;27.16)	18.39 (15.54;20.61)	- 3.58 (- 16%)	0.21
Q_Quant_ (ml/min/100 ml)	339 (212;457)	246 (197;311)	- 93 (- 27%)	0.03*
QDP (%)	8.15 (7.09;11.34)	5.04 (3.16;6.99)	- 3.11 (- 38%)	≤0.01*
VDP (%)	4.39 (2.70;6.91)	3.36 (0.26;5.54)	- 1.03 (- 23%)	0.21
VQM (%)	88.78 (84.02;89.20)	91.82 (91.01;94.03)	- 3.04 (- 3.4%)	≤0.01*

Values are displayed as median with the first and third quartile (Q1;Q3). Significant results are marked with *.

RVent: Regional ventilation; Q: Perfusion; Q_Quant_: Quantified perfusion; QDP: Perfusion defect percentage; VDP: Ventilation defect percentage; VQM: Ventilation perfusion match

Table 3 discloses a significant increase of the median skewness (1.94 vs. 2.53) and kurtosis (8.31 vs. 11.42) of the Q-values at 3T. Fig 4 displays the increasing skewness and kurtosis of the Q- and RVent-values of all participants one histogram analysis.

**Table 3:** Skewness and kurtosis of Q and RVent of all participants.

	Skewness and kurtosis of Q and RVent			Wilcoxon signed-rank test
	1.5 Tesla	3 Tesla	Difference	*P*-value
Skewness Q	1.94 (1.44;2.12)	2.53 (2.12;2.82)	+ 0.59 (+ 30%)	≤0.01*
Kurtosis Q	8.31 (6.87;10.53)	11.42 (9.24;15.25)	+ 3.11 (+ 37%)	≤0.01*
Skewness V	0.02 (-0.21;0.65)	0.38 (0.06;1.86)	+ 0.36 (+ 1800%)	0.08
Kurtosis V	4.00 (2.88;5.88)	5.46 (2.77;17.64)	+ 1.46 (+ 37%)	0.41

Values are displayed as median with the first and third quartile (Q1;Q3). Significant results are marked with *.

As demonstrated in Table 4, neither the CNR nor the SNR increased significantly at 3T.

**Table 4:** SNR and CNR results of all participants.

	Signal and Contrast to Noise Ratio			Wilcoxon signed-rank test
	1.5 Tesla	3 Tesla	Difference	*P*-value
SNR Expiration	10.01 (7.22;12.30)	8.47 (7.53;10.74)	- 1.54 (- 15%)	0.74
SNR Inspiration	7.33 (5.63;9.02)	6.56 (5.11;8.00)	- 0.77 (- 11%)	0.48
CNR Expiration	7.96 (7.10;14.66)	15.55 (8.99;21.65)	+ 7.59 (+ 95%)	0.23
CNR Inspiration	7.86 (6.39;14.76)	13.12 (9.03;18.98)	+ 5.26 (+ 67%)	0.14

Values are displayed as median with the first and third quartile (Q1;Q3).

### Bland-Altman analysis

**Fig 5:**
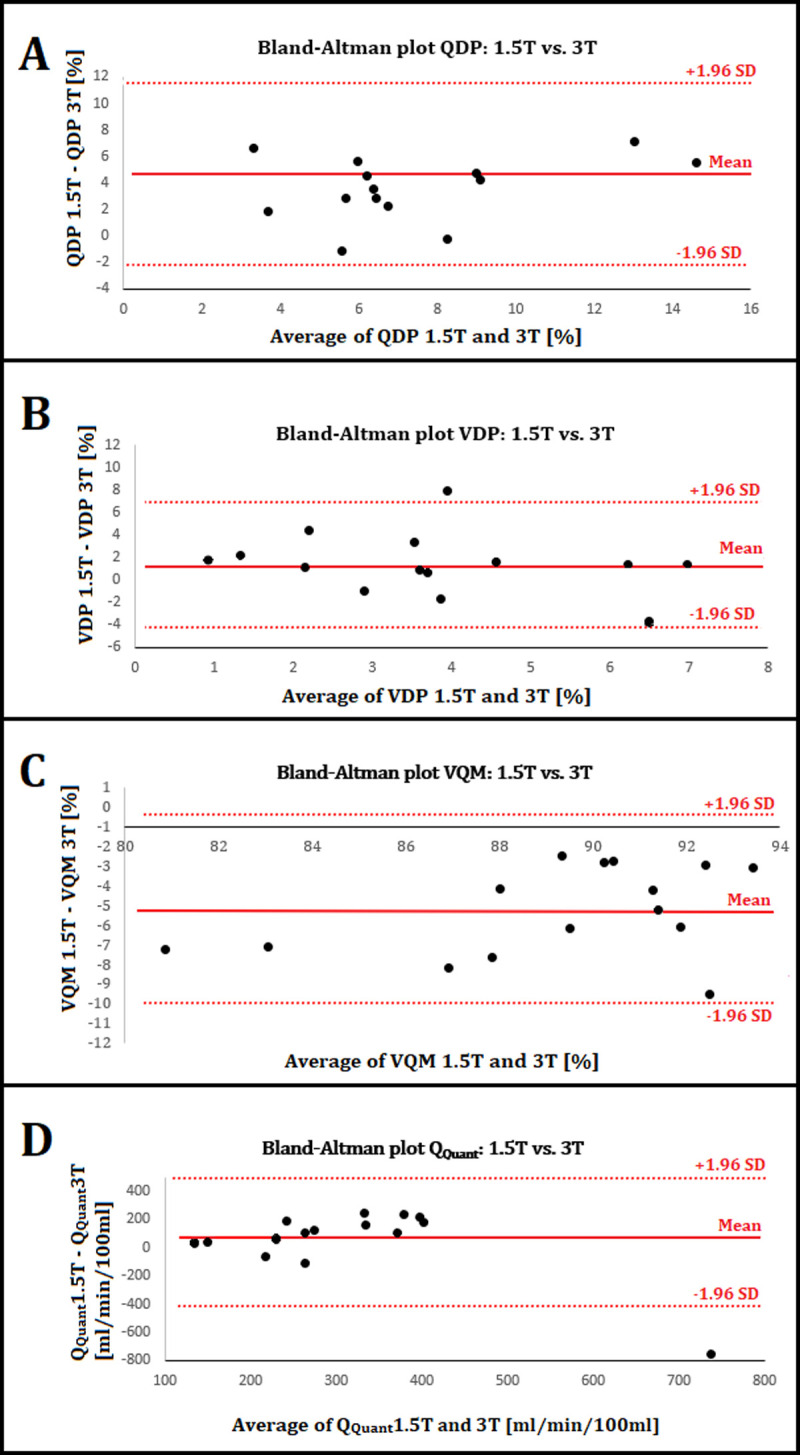
Bland-Altman plots. **A)** The mean average of QDP ((QDP 1.5T –QDP 3T)/2) is 7.39% and the mean difference (QDP 1.5T –QDP 3T) (solid red lines) is 4.29% with a SD of 3.54%. The upper and lower 95% confidence intervals mean ± 1.96*SD (dashed red lines) are 11.23% and -2.64%. **B)** The mean average of VDP is 4.04% and the mean difference is 1.23% with a SD of 2.76%. The confidence intervals are 6.64% and -4.18%. **C)** The mean average of VQM is 89.44% and the mean difference is -5.15% with a SD of 2.45%. The confidence intervals are -0.35% and -9.95%. **D)** The mean average of Q_Quant_ is 314 ml/min/100ml and the mean difference is 54 ml/min/100ml with a SD of 246 ml/min/100ml. The confidence intervals are 537 and -428 ml/min/100ml.

The mean differences were 4.29% for QDP, 1.23% for VDP, -5.15% for VQM and 54 ml/min/100ml for Q_Quant_ in the Bland-Altman analysis (see Fig 5). A minimal sample size of 12 subjects is necessary to detect significant differences in QDP and VQM according to our retrospective sample size calculation.

### Sørensen-Dice and VQ-maps

The mean Sørensen-Dice coefficients depicting the spatial overlap between both series were 0.91 for QDP, 0.94 for VDP and 0.87 for VQM using the above-mentioned thresholds. Median values of the binary maps QDP and VDP are displayed in Table 2.

## Discussion

The first main finding of our study is, that neither the parameters RVent, Q and VDP, nor the SNR or the CNR altered significantly at 3T. Despite marked absolute and relative differences of the functional parameters, no significance was shown. As presented in Table 4, significance is not directly dependent on relative or absolute differences, but rather on the value distribution and standard deviation respectively. The median SNR during the expiratory state ranges between 1.5T and 3T from 10.01 to 8.47, which is in good agreement with previous reports of Yu et al. [32]. Also, the up to 95% increase of CNR was not significant. The higher field strength and advanced hardware of the 3T scanner were used to increase the temporal resolution by minimizing TR and TE rather than to improve SNR and CNR.

The second main finding of our study is the significant 27% decrease of Q_Quant_ at 3T. In a previous study, the opposite effect was shown after the administration of gadolinium based contrast agents [19]. As a well-known effect of higher field strengths is an increase of T1 relaxation time of blood in the lungs, it is not surprising to see correspondingly lower values for Q_Quant_ in this study [21, 33].

The third main finding of our study is the significant 38% decrease of QDP at 3T, although we were using the same relative thresholds based on the median and the standard deviation. Significant increases of the skewness and the kurtosis confirmed our assumption of a different distribution of the Q-values at 3T (Fig 4). The mean R^2^ of the piecewise fits of the Q-values to the sine function decreased not significantly from 0.55 at 1.5T to 0.45 at 3T (*P* = 0.11). Nevertheless, there is high spatial agreement between the QDP maps and also between the VDP maps of 1.5T and 3T with Sørensen-Dice coefficients of > 0.9 with the used thresholds. This is because differing nuances of the value distribution between 1.5T and 3T are not considered in binary maps like QDP and VDP and because there were very few defect areas in healthy participants at the used thresholds.

According to the before mentioned increase of T1, T2* decreases more than T2 due to higher field inhomogeneities, susceptibility effects and spin-spin interactions [20, 34, 35]. Again, this could be a potential cause for the differing distributions of the Q- and RVent-values–expressed by significant increases of the skewness and the kurtosis–at 3T.

Beside the earlier mentioned characteristics of lung tissue with low intrinsic signal and high susceptibility artifacts, the two-compartment model of extravascular lung water (EVLW) and intravascular lung water (IVLW) in the human lung could be a possible explanation for different signal alteration of Q-maps and RVent-maps at higher field strengths. Triphan et al. explain, that T1 is not only highly variable, but also, that both compartments of the lung tissue contain different values of T1 and T2*. Assuming a relatively slow proton exchange compared to the relaxation times of the compartments, long TEs are predominantly measuring intravascular protons, whereas short TEs are measuring the extravascular compartment [36]. Likewise, the variability of T2* between subjects is very high [37], but may be negligible during tidal breathing [32]. Bydder et al. point out that all tissues have multicomponent T2s resulting of a mixture of different T2 components [38]. In this regard, it is important to understand the nonlinear relationship between relaxation rate and field strength [32, 39]. This would explain a differing sensibility of perfusion- and ventilation-maps on field strength, as their signal is based on intravascular flow related enhancement (Q-map) or combined intra- and extravascular lung density changes (RVent-map).

Possible improvements at higher field strengths could be achieved by the use of modified imaging sequences in regards to the SAR limits, artifacts and field inhomogeneities, the image contrast and higher SNR [39]. Rotärmel et al. already demonstrated the advantages of steady-state free precession (SSFP) over the GRE Fast Low Angle Shot (FLASH) sequence regarding SNR and lung-vessel-contrast at 1.5T [40].

In this context the results of Bauman et al. are very promising. Whereas a balanced steady-state free precession (bSSFP) sequence is sensitive to artifacts and endangers SAR limits by using high flip angles, the proposed transient spoiled gradient echo (tSPGR) acquisitions provided a significant increase in SNR to contemporary spoiled gradient recalled (SPGR) acquisitions at 3T [24].

The main limitation of our study is the difference of the used sequence parameters–flip angle, TE and TR–between 1.5T and 3T. The respective functionality of the sequences at 1.5T and 3T is given, but any modification of the sequence would entail a reevaluation of the functional parameters to guarantee comparability. For instance a smaller flip angle of 4° was used at 3T to compensate for the increasing T1 of blood at 3T [41]. Additionally, the advanced hardware with differing coil design of the 3T Skyra allows for higher slew rates of up to 200 mT/m/ms leading to higher imaging speed and temporal resolution.

A further limitation of our study is the lack of more established reference standards such as 129-Xe ventilation MRI and dynamic contrast enhanced perfusion MRI as gold standards [42, 43]. By having a group of healthy participants, it is very difficult to set a comprehensive threshold for QDP and VDP. Although these parameters have proven to be highly valuable in various studies with patients [12, 17, 44], there is limited interstudy comparability, because their thresholds were set differently according to the underlying data. As healthy participants only have rare perfusion and ventilation defect areas, it is not surprising to achieve Dice-values of over 0.9. Additionally, FD derived functional parameters have shown to exhibit good, but still limited reproducibility [19, 45, 46]. In particular perfusion quantification is influenced by various technical and physiological circumstances, like field inhomogeneities, physiological breathing and perfusion variability, inaccuracy of the estimated receive coil sensitivities or the angle of blood flow towards the imaging plane [47]. For example, no randomization of the 1.5T and 3T scanner was done. By this, participants could have been more relaxed during the second scan already knowing the procedure.

Beside RVent and Q_Quant_, more fully quantified functional parameters need to be developed in further studies to achieve optimal comparability and their dependency on varying relaxation times needs to be considered. Ideally, a multicompartment mapping of PD, T1 and T2 would be helpful, but has not been developed or published yet. The functional parameters could potentially be adapted to these tissue characteristics.

## Conclusion

RVent, Q, VDP, SNR and CNR were not altered significantly at the used parameters. Significant differences in QDP may be related to the field strength. Q_Quant_ reveals only poor reproducibility between 1.5T and 3T. Healthy participants with minimal defects present high spatial agreement of QDP and VDP.

## Supporting information

S1 Dataset Dataset of all results. (XLSX)
